# Large Pericardial Effusion As the Initial Presentation of Mixed Connective Tissue Disease in a Severely Malnourished Patient: A Diagnostic Challenge

**DOI:** 10.7759/cureus.110681

**Published:** 2026-06-11

**Authors:** Mohammed N Taha, Moath Al-Shudifat

**Affiliations:** 1 Internal Medicine, Baptist Health Medical Center-North Little Rock, North Little Rock, USA

**Keywords:** anti-u1 ribonucleoprotein antibodies, autoimmune pericarditis, cardiac tamponade, hypoalbuminemia, hypothyroidism, malnutrition, mixed connective tissue disease, pericardial effusion, pericardiocentesis

## Abstract

Pericardial effusion, defined as the abnormal accumulation of fluid within the pericardial sac, has a broad differential diagnosis, and identifying the underlying etiology can be particularly challenging when multiple potential causes coexist. Mixed connective tissue disease (MCTD) is a rare systemic autoimmune disorder. MCTD is characterized by overlapping features of systemic lupus erythematosus (SLE), systemic sclerosis (SSc), and polymyositis in association with anti-U1 ribonucleoprotein antibodies (anti-U1-RNP). Although pericarditis is the most common cardiac manifestation of MCTD, large pericardial effusion as the initial presenting feature has been described in only a small number of case reports. We describe the case of a 62-year-old malnourished female (BMI 17.7 kg/m^2^) with a history of hypertension, hypothyroidism, and chronic dysphagia who presented with persistent nausea and vomiting. Laboratory evaluation revealed leukopenia, anemia, hypoalbuminemia (2.8 g/dL), subclinical hypothyroidism, and normal inflammatory markers. CT obtained for gastrointestinal symptoms incidentally revealed a large pericardial effusion. Transthoracic echocardiography confirmed a large circumferential effusion with an end-diastolic diameter of 4.87 cm and early tamponade physiology, including right atrial systolic collapse and significant respiratory variation in tricuspid inflow. Ultrasound-guided pericardiocentesis yielded approximately 1000 mL of clear yellow fluid; cultures and cytology were negative. The sausage-shaped swollen fingers seen clinically prompted serologic tests, which were positive for antinuclear antibodies (ANA), anti-U1-RNP, and anti-Sjögren's-syndrome-related antigen A (anti-SS-A) antibodies. Anti-double-stranded DNA (anti-dsDNA), anti-Smith, anti-Scl-70, and anti-centromere antibodies were negative. Esophagogastroduodenoscopy showed a lower esophageal stricture consistent with chronic reflux. The patient's findings were consistent with the Kasukawa classification criteria for MCTD, with the patient demonstrating anti-U1-RNP antibodies, swollen digits (a common symptom), pericarditis/serositis (an SLE-like feature), and esophageal involvement (an SSc-like feature). The patient improved after pericardiocentesis and nutritional optimization and was discharged with multidisciplinary follow-up. This case illustrates the diagnostic challenge of determining the etiology of a large pericardial effusion in a patient with multiple possible confounding factors, including malnutrition, subclinical hypothyroidism, and unrecognized autoimmune disease. Normal CRP and absence of fever were suggestive of a noninflammatory process, which may be seen in autoimmune pericardial disease, though a normal CRP is not specific for autoimmune pericarditis. While prior case reports have described large pericardial effusion as an initial manifestation of MCTD, the unique contribution of this case lies in the specific diagnostic challenge of disentangling MCTD from concurrent malnutrition and subclinical hypothyroidism in an elderly patient, a clinical scenario that underscores the importance of maintaining a high index of suspicion for autoimmune disease in patients with idiopathic pericardial effusions, even when nutritional or metabolic causes seem more likely.

## Introduction

Pericardial effusion is the abnormal accumulation of fluid in the pericardial sac, defined as the accumulation of >50 mL of fluid [1]. There are many causes of pericardial effusion, and idiopathic effusion comprises more than half of all cases. Other causes are infectious, malignant, traumatic, metabolic, and autoimmune diseases [2]. Differentiating the etiology may be difficult in patients with overlapping features.

It's critical to distinguish the underlying pathophysiology of pericardial disease, as this guides treatment selection [2]. The inflammatory phenotype is characterized by activation of the inflammasome and release of cytokines and is associated with leukocytosis and increased inflammatory markers such as C-reactive protein (CRP) [1]. The non-inflammatory phenotype of autoimmune diseases is typically characterized by a normal or slightly elevated CRP [1].

Mixed connective tissue disease (MCTD) is a systemic autoimmune disorder first described in 1972 [3]. MCTD is the least common systemic connective tissue disease, with a point prevalence of 3.8 per 100,000 and a mean annual incidence of 2.1 per million per year in population-based data from Norway [4]. The disease is characterized by the presence of overlapping clinical features of several connective tissue diseases, especially systemic lupus erythematosus (SLE), systemic sclerosis (SSc), and polymyositis, in association with high titers of anti-U1 ribonucleoprotein antibodies (anti-U1-RNP) [5]. MCTD involves multiple organ systems, and cardiovascular involvement is found in a substantial number of patients. Pericarditis and pericardial effusion are the most frequently described cardiac manifestations of the disease, with 30% and 43% prevalence in two prospective studies, while cardiac tamponade or constrictive pericarditis is rare, reported in only 3.6% of patients [6].

Pericardial effusion size is determined by the greatest end-diastolic diameter on echocardiography. Large effusion is categorized with a diameter >2 cm [1]. Although small pericardial effusions are relatively common in systemic autoimmune disorders, the occurrence of large effusions is rare and usually preceded by other disease manifestations, but few case reports have described it as the initial manifestation of connective tissue disease [7]. Pericardial effusion in the malnourished geriatric population is poorly characterized, with a lack of large prospective studies, but has been linked to hypoalbuminemia in patients with conditions such as chronic kidney disease and cardiac amyloidosis [8,9].

This report describes a case of a severely malnourished elderly patient who presented with gastrointestinal symptoms and was incidentally found to have a large pericardial effusion. This case underscores the challenges in distinguishing nutritional and autoimmune etiologies of pericardial effusion and the need for a comprehensive evaluation in the setting of idiopathic pericardial disease.

## Case presentation

A 62-year-old female with a past history of hypertension, hypothyroidism, chronic dysphagia, and progressive weight loss presented to the hospital with a complaint of persistent nausea and vomiting worsening over the past two to three weeks. She had been admitted to the hospital multiple times for similar symptoms but had no prior outpatient follow-up with a gastroenterologist.

The patient was frail and cachectic, with a body mass index of 17.7 kg/m^2^ and an albumin of 2.8 g/dL. She met the Global Leadership Initiative on Malnutrition (GLIM) criteria for Stage 2 (severe) malnutrition based on a phenotypic criterion of low BMI (<18.5 kg/m^2^ for age <70 years) and an etiologic criterion of reduced food intake due to chronic dysphagia and recurrent vomiting [10]. Vital signs were stable except for tachycardia. The review of systems was remarkable for generalized weakness.

Laboratory tests revealed leukopenia, anemia, and mild hyponatremia. Lipase, troponin, procalcitonin, magnesium, lactate, and inflammatory markers, including CRP, were all within normal ranges. Thyroid function tests showed an increased thyroid-stimulating hormone (TSH) level with normal free thyroxine levels (Table 1).

**Table 1:** Initial hematologic, metabolic, inflammatory, and endocrine laboratory findings. IU/mL: international units per milliliter; K/cm^3^: thousand cells per cubic millimeter; g/dL: grams per deciliter; mEq/L: milliequivalents per liter; ng/mL: nanograms per milliliter; ng/L: nanograms per liter; mmol/L: millimoles per liter Values shown in bold indicate abnormal laboratory results.

Test	Result	Reference Range	Interpretation
White blood cell count	2.2	4.5-11.0 K/cm^3^	Low
Hemoglobin	8.3	12.0-16.0 g/dL	Low
Platelet count	199	150-400 K/cm^3^	Normal
Sodium	134	136-145 mEq/L	Mild hyponatremia
Potassium	4.8	3.5-5.1 mEq/L	Normal
Creatinine	0.74	0.57-1.11 mg/dL	Normal
Albumin	2.8	3.5-5.2 g/dL	Low
C-reactive protein	0.22	0.00-0.50 mg/dL	Normal
Procalcitonin	0.04	0.00-0.05 ng/mL	Normal
High-sensitivity troponin	7.7	<14.0 ng/L	Normal
Lactate	1.3	0.5-2.0 mmol/L	Normal
Thyroid-stimulating hormone	12.747	0.350-4.940 IU/mL	Elevated
Free thyroxine (Free T4)	1.16	0.70-1.48 ng/dL	Normal

**Figure 1:**
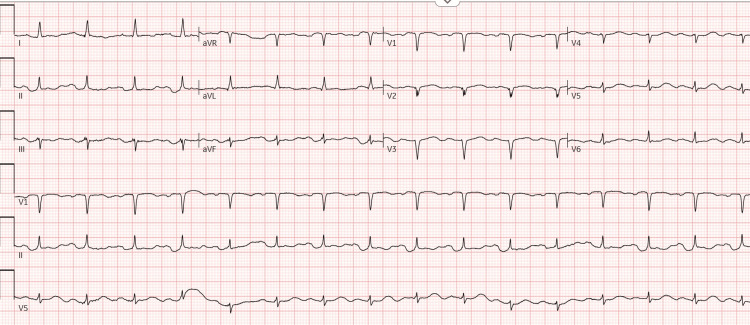
Electrocardiogram demonstrating sinus rhythm with low-voltage QRS complexes.

Electrocardiography demonstrated sinus rhythm with first-degree atrioventricular block and low-voltage QRS complexes (Figure 1), a common finding in the setting of a large pericardial effusion due to electrical dampening by the surrounding fluid [11].

**Figure 2:**
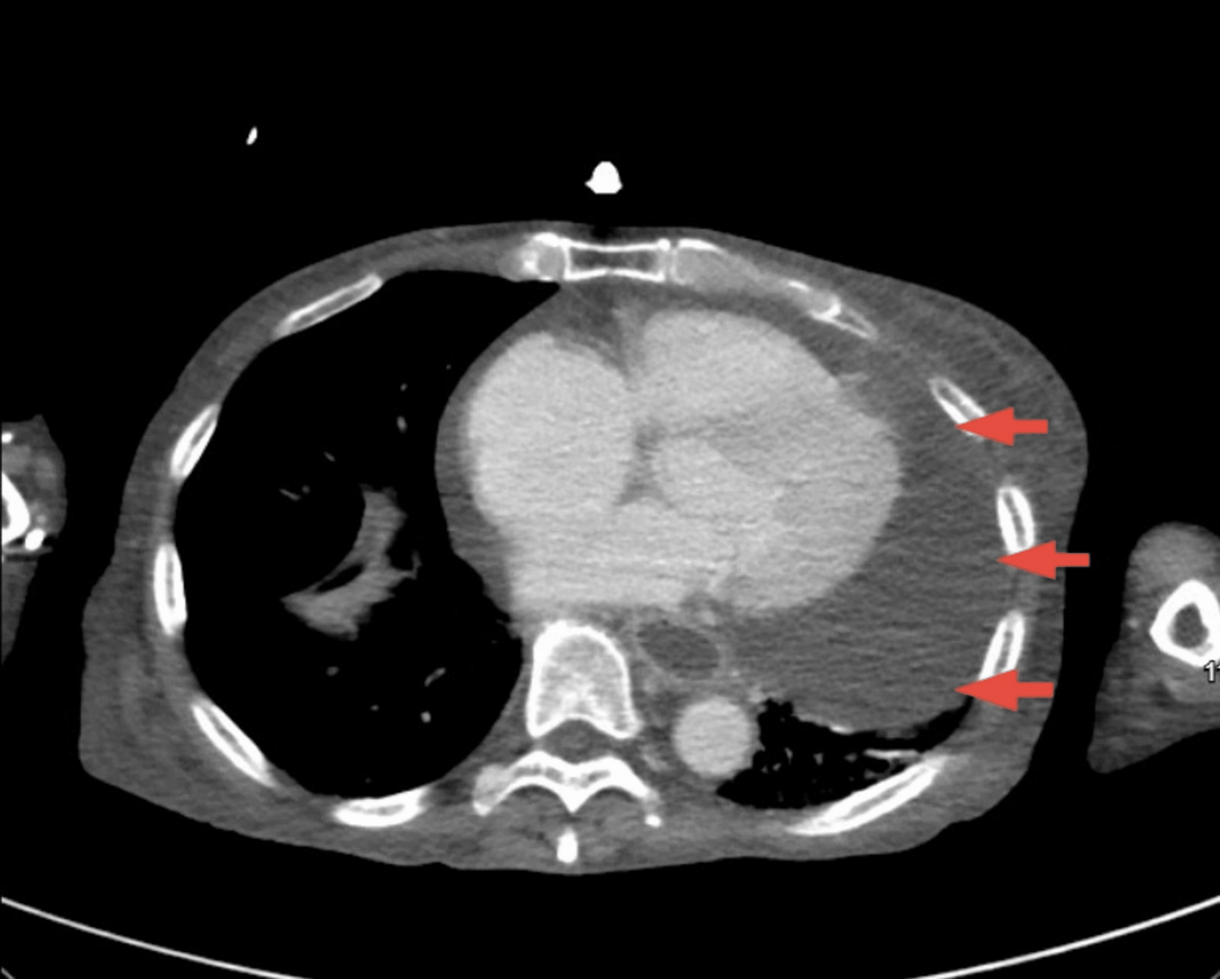
Computed tomography demonstrating a large circumferential pericardial effusion (arrows).

Computed tomography of the abdomen and pelvis obtained during evaluation of gastrointestinal symptoms revealed a large pericardial effusion (Figure 2), along with esophageal stricture and findings concerning for superior mesenteric artery (SMA) syndrome. The CT finding of possible SMA syndrome was attributed to the patient's severely malnourished state, as loss of mesenteric fat narrows the aortomesenteric angle and predisposes to compression of the third portion of the duodenum. This finding may have contributed to the patient's recurrent nausea and vomiting. Conservative management with nutritional rehabilitation was pursued.

**Figure 3:**
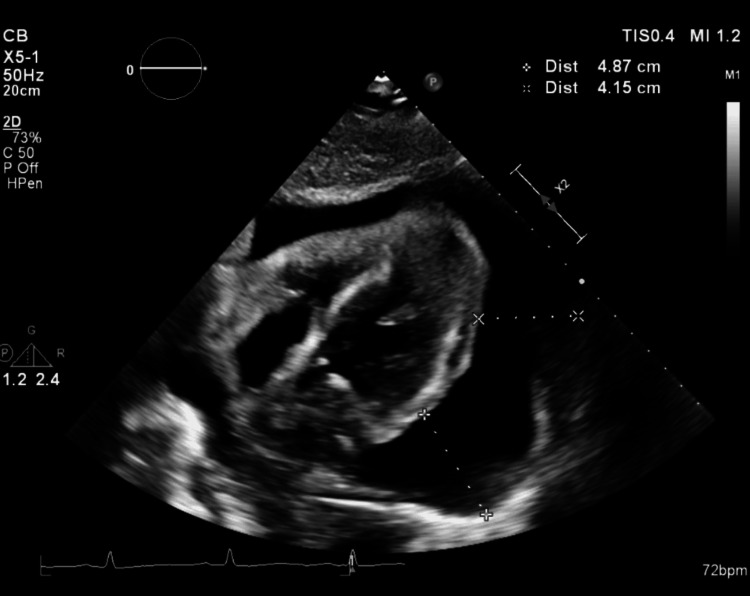
Transthoracic echocardiography demonstrates a large pericardial effusion with right atrial systolic collapse.

**Figure 4:**
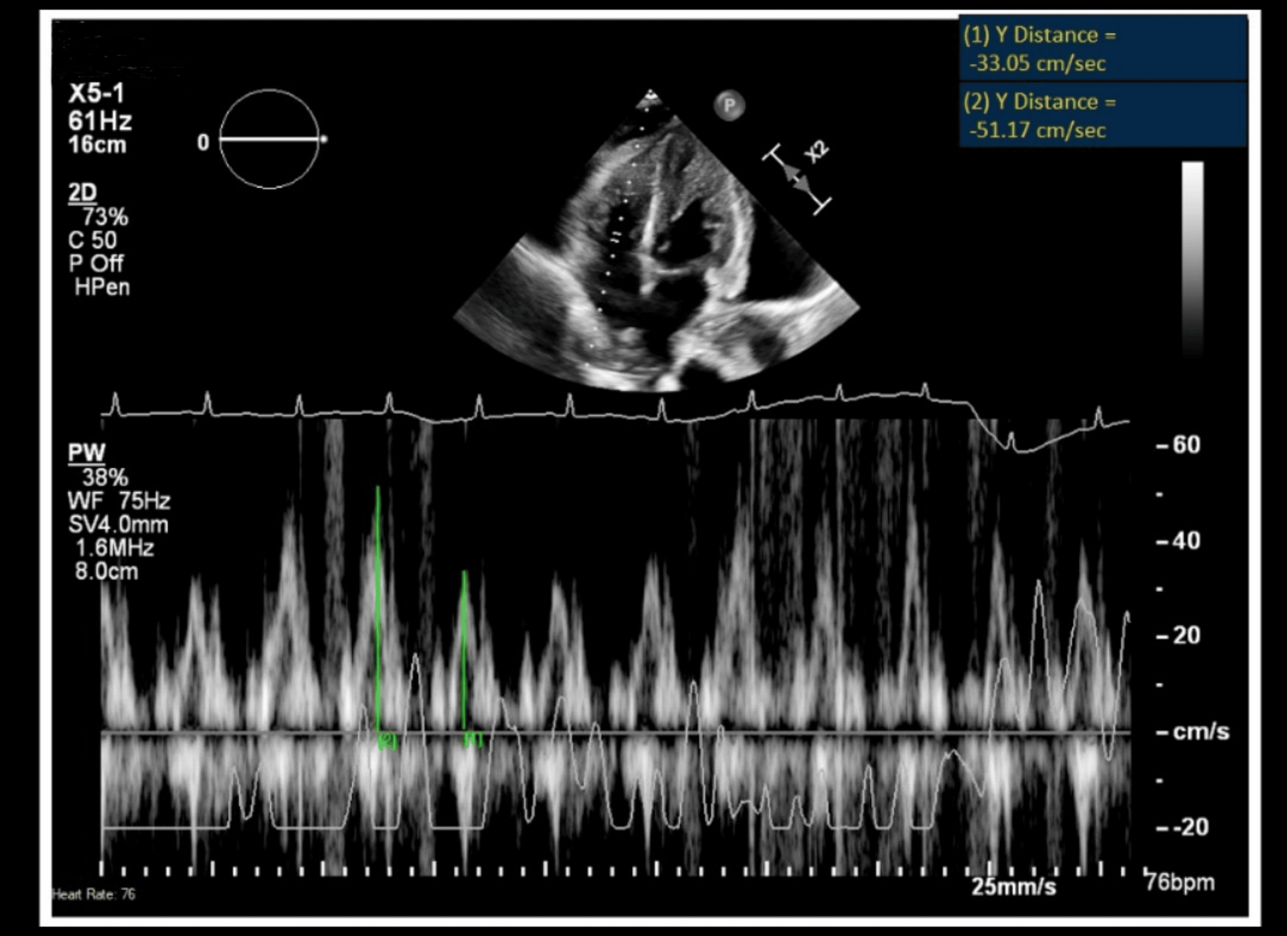
Pulsed-wave Doppler of tricuspid inflow demonstrating significant respiratory variation.

**Figure 5:**
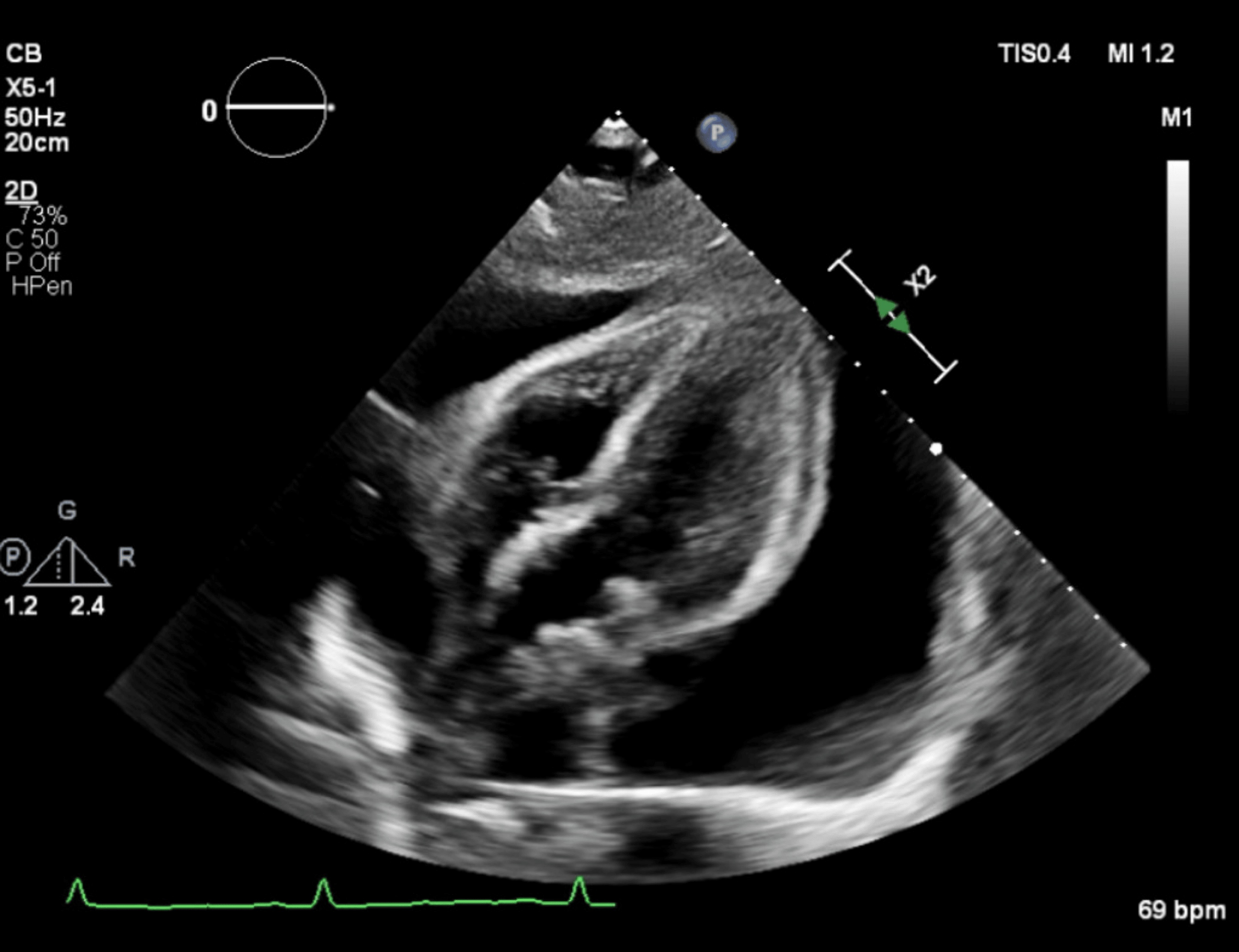
Apical four-chamber echocardiographic view demonstrating a large circumferential pericardial effusion with right atrial systolic collapse suggestive of early tamponade physiology, without right ventricular diastolic collapse.

**Figure 6:**
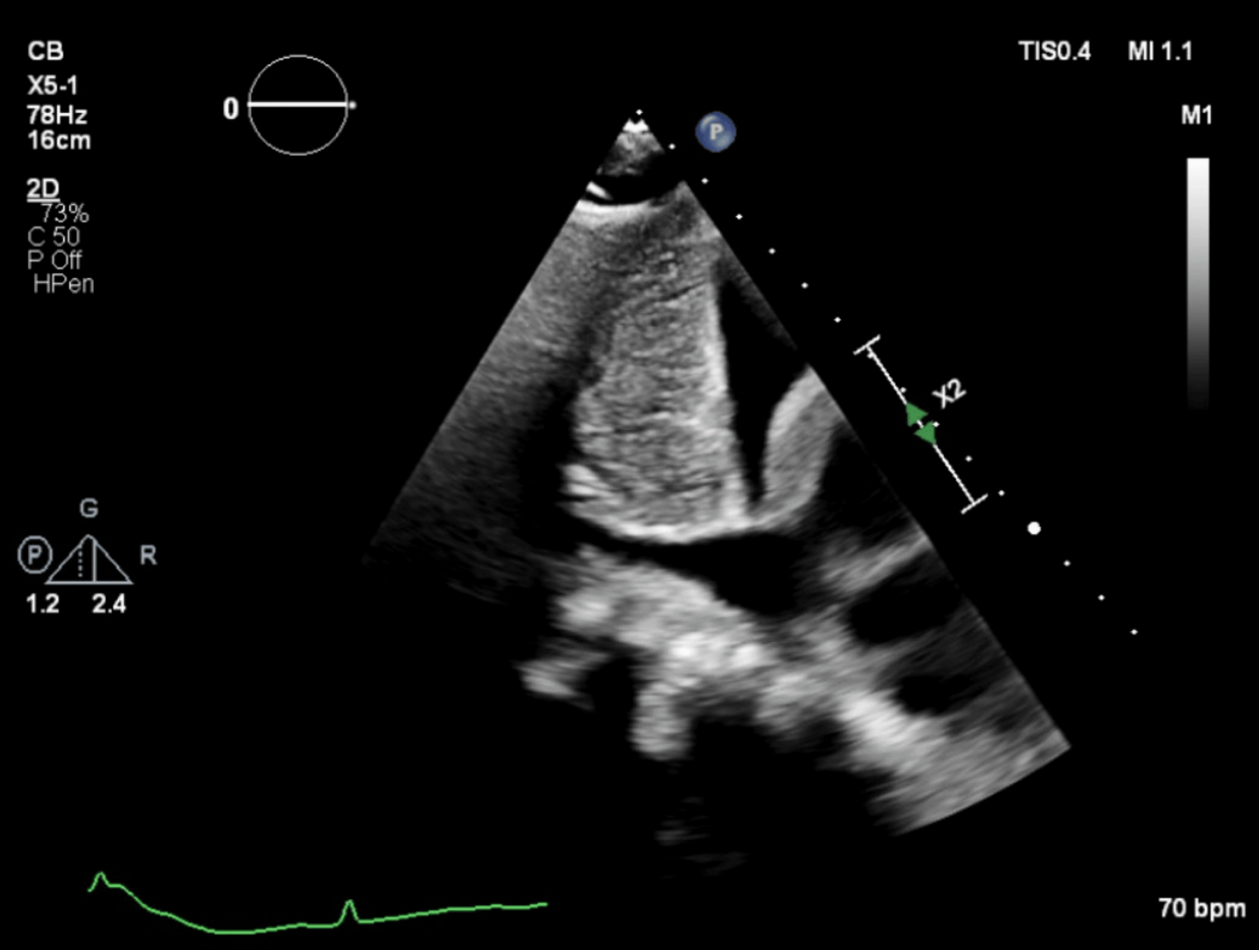
Subcostal two-dimensional echocardiographic view demonstrating a large pericardial effusion and small collapsible inferior vena cava.

Transthoracic echocardiography confirmed the presence of a large circumferential pericardial effusion with the greatest end-diastolic diameter of 4.87 cm (Figure 3). Doppler analysis demonstrated significant respiratory variation in tricuspid inflow (Figure 4) and mild right atrial systolic collapse (Figure 5), findings suggestive of early tamponade physiology. However, there was no right ventricular diastolic collapse, and the inferior vena cava remained small and collapsible (Figure 6).

Ultrasound-guided pericardiocentesis was performed because of the large size of the effusion and concern for possible progression due to early signs of tamponade. We evacuated approximately 1000 milliliters of clear yellow fluid.

Pericardial fluid cultures were negative for bacterial or fungal growth, and cytology was negative for malignant cells. Pericardial fluid cell count and differential, protein, lactate dehydrogenase (LDH), and glucose were not obtained, which is acknowledged as a limitation of the diagnostic evaluation.

While in the hospital, the patient was found to have sausage-shaped swollen fingers, suggesting an underlying autoimmune disease. Additional serologic studies were positive for antinuclear antibodies (ANA), anti-U1-RNP, and anti-Sjögren's-syndrome-related antigen A (anti-SS-A) antibodies. Anti-double-stranded DNA (anti-dsDNA), anti-Smith, anti-Scl-70, and anti-centromere antibodies were negative (Table 2). The anti-U1-RNP result was obtained using a U1-RNP-specific enzyme-linked immunosorbent assay (ELISA), reported in antibody index (AI) units.

**Table 2:** Autoimmune serologic studies. ANA: anti-nuclear antibody; anti-dsDNA: anti-double-stranded DNA antibody; anti-SS-A: Sjögren syndrome-related antigen A antibody; anti-SS-B: Sjögren syndrome-related antigen B antibody; anti-U1-RNP: anti-U1 ribonucleoprotein antibody; anti-Scl-70: anti-topoisomerase I antibody; anti-Jo-1: anti-histidyl transfer RNA synthetase antibody; AI: antibody index; IU/mL: international units per milliliter Values shown in bold indicate abnormal laboratory results.

Test	Result	Reference Range	Interpretation
ANA screen	Positive	Negative	Positive
Anti-dsDNA antibody	1 IU/mL	≤4 IU/mL	Negative
Anti-SS-A antibody	1.2 AI	<1.0 AI	Positive
Anti-SS-B antibody	<1.0 AI	<1.0 AI	Negative
Anti-Smith antibody	<1.0 AI	<1.0 AI	Negative
Anti-U1-RNP antibody	>8 AI	<1.0 AI	Positive
Anti-centromere B antibody	<1.0 AI	<1.0 AI	Negative
Anti-Scl-70 antibody	<1.0 AI	<1.0 AI	Negative
Anti-Jo-1 antibody	<1.0 AI	<1.0 AI	Negative

**Figure 7:**
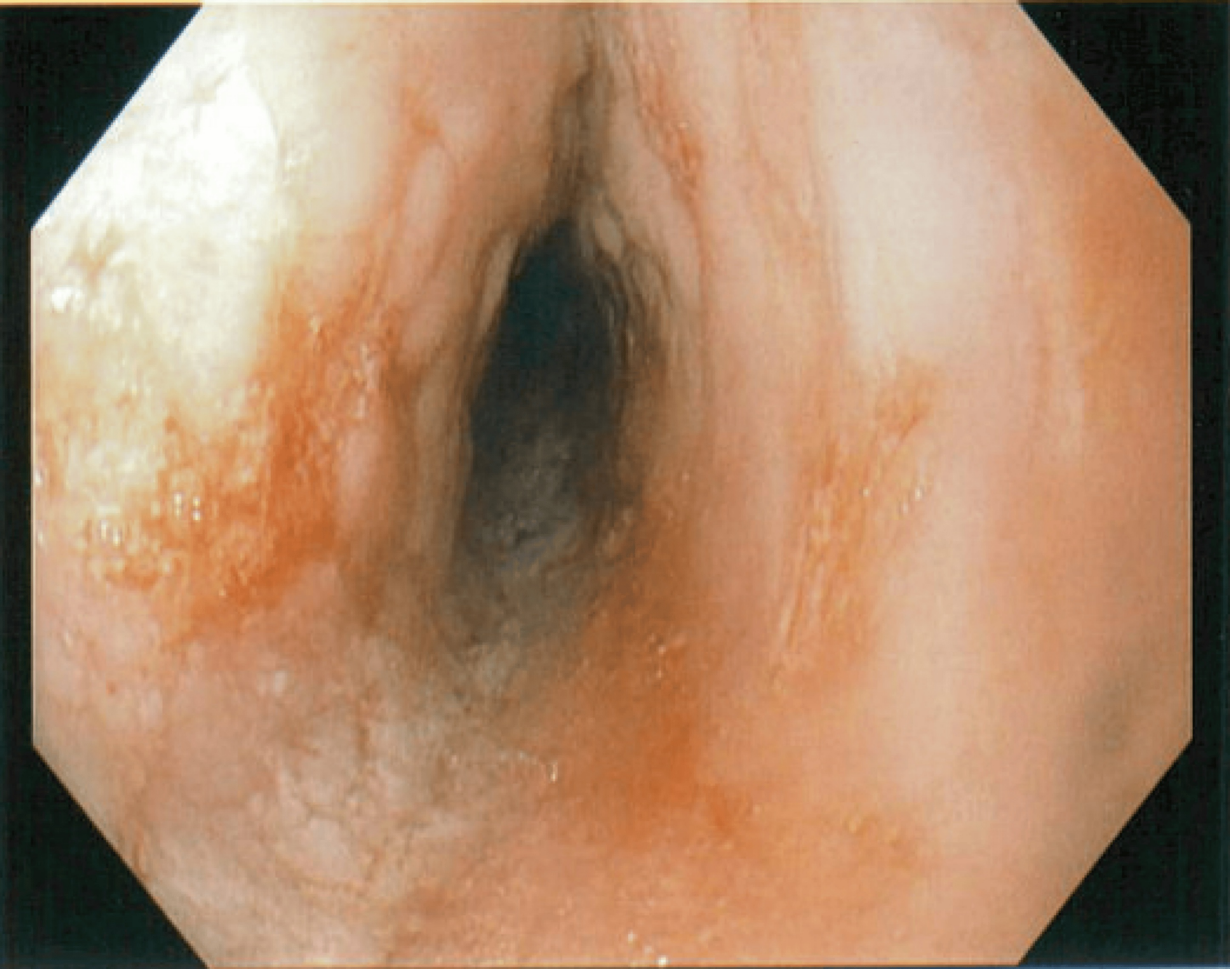
Esophagogastroduodenoscopy (EGD) showing esophageal stricture.

Gastroenterology was consulted for evaluation of recurrent vomiting and dysphagia. Esophagogastroduodenoscopy (EGD) revealed a stricture in the lower third of the esophagus due to chronic acid reflux. Biopsy revealed benign squamous mucosa with ulceration and no malignancy (Figure 7).

The patient's findings were consistent with the Kasukawa classification criteria for MCTD: anti-U1-RNP antibodies were present as the required serologic marker; swollen fingers fulfilled the common symptom criterion; pericarditis with large pericardial effusion satisfied the SLE-like disease category; and esophageal involvement satisfied the SSc-like disease category [6,12].

The patient was treated for malnutrition with nutritional supplements and multivitamin therapy. Levothyroxine replacement therapy was initiated for subclinical hypothyroidism. After pericardiocentesis and EGD, she had a significant improvement in symptoms and was able to tolerate oral intake. She was discharged stable with follow-up arranged with cardiology, gastroenterology, and rheumatology. At three-month cardiology follow-up, the patient remained clinically stable without symptoms suggestive of recurrent pericardial effusion. Repeat echocardiography was recommended but was not performed per patient preference. Follow-up with rheumatology and gastroenterology had not yet occurred at the time of this report.

## Discussion

This case illustrates the difficulty in determining the etiology of a large pericardial effusion in an elderly patient with severe malnutrition and several other potential confounding etiologies, including malnutrition, hypothyroidism, and an unrecognized systemic autoimmune disease. The incidental finding of a large pericardial effusion during work-up for gastrointestinal symptoms and the eventual identification of MCTD as the most likely underlying etiology emphasizes the necessity of a broad differential diagnosis and extensive serologic work-up in patients with idiopathic pericardial effusions.

Pericardial effusion has a broad spectrum of underlying etiologies, and it is difficult to differentiate between them, especially when there are multiple potential etiologies. This patient had three possible etiologies: autoimmune pericardial disease, hypothyroidism, and malnutrition-related hypoalbuminemia. The 2025 American College of Cardiology (ACC) Expert Consensus Statement on Pericarditis advocates a stepwise approach to the triage of pericardial effusions, beginning with assessment for hemodynamic compromise, followed by assessment of inflammatory markers, workup for specific associated conditions, and consideration of the size and duration of the effusion [1].

The distinction between inflammatory and non-inflammatory phenotypes of pericardial disease is of clinical importance, as it directly affects the choice of treatment [1]. In the inflammatory phenotype, which comprises 80-90% of cases of pericarditis, activation of the NOD-like receptor family pyrin domain-containing 3 (NLRP3) inflammasome is a key feature, and an elevated CRP is a hallmark of the disease, often with fever and leukocytosis [1]. The noninflammatory phenotype, seen in 10-20% of cases, is commonly associated with autoimmune diseases and usually has a normal or near-normal CRP [1]. In this patient, normal CRP and lack of fever were suggestive of a noninflammatory process, which may be seen in autoimmune pericardial disease, though a normal CRP does not exclude other etiologies and is not specific for autoimmune pericarditis.

Pericardial effusion is a recognized complication of hypothyroidism and has been reported in 3-37% of hypothyroid patients [13]. The mechanism involves increased permeability of epicardial vessels and decreased lymphatic drainage of albumin, leading to accumulation of fluid in the pericardial space [13]. The elevated TSH level (12.7 mIU/L) with normal free thyroxine levels was consistent with subclinical hypothyroidism. Levothyroxine replacement therapy was initiated during hospitalization. Although subclinical hypothyroidism can be associated with pericardial effusion, large effusions with hemodynamic compromise are characteristically reported in severe, overt hypothyroidism with markedly elevated TSH and low free thyroxine levels, rather than in subclinical disease [13]. In a classic echocardiographic study of myxedematous patients, pericardial effusions were present in 30%, but none developed tamponade, and all effusions resolved with thyroxine replacement [14]. The subclinical nature of the thyroid dysfunction in this patient, combined with the large volume of the effusion and early tamponade physiology, made hypothyroidism an unlikely sole explanation for the pericardial effusion.

Malnutrition and hypoalbuminemia are other possible causes of pericardial effusion in this patient. Hypoalbuminemia decreases plasma oncotic pressure, promoting fluid shift from the intravascular to the extravascular spaces, including the pericardial space [15]. In patients with chronic kidney disease, hypoalbuminemia has been demonstrated to be an independent predictor of moderate to severe pericardial effusion [9]. However, the development of hypoalbuminemia secondary to malnutrition is usually a slow process as opposed to the rapid changes seen with inflammatory redistribution [15]. In addition, effusions driven solely by reduced oncotic pressure would be expected to accumulate slowly and remain hemodynamically insignificant, as the pericardium can accommodate gradual fluid accumulation with minimal increase in intrapericardial pressure [1]. The presence of early tamponade physiology in this patient, with right atrial collapse in systole and marked respiratory variation in tricuspid inflow, suggested a process beyond simple transudation.

The CT finding concerning for SMA syndrome warrants discussion, as it may have contributed to the patient's recurrent vomiting and further exacerbated her malnutrition. SMA syndrome results from compression of the third portion of the duodenum between the aorta and the SMA, typically precipitated by significant weight loss and reduced mesenteric fat [16]. Initial management is conservative, with nutritional rehabilitation aimed at restoring mesenteric fat and widening the aortomesenteric angle [17]. The patient's gastrointestinal symptoms were likely multifactorial, with contributions from the esophageal stricture and possible SMA syndrome. SMA syndrome was managed conservatively with nutritional rehabilitation and outpatient follow-up.

A key clinical observation leading to serologic evaluation for autoimmune disease was the finding of sausage-shaped swollen fingers during hospitalization. Among the most characteristic clinical features of MCTD are puffy or swollen fingers, which are included in several sets of diagnostic criteria [12,18]. Serologic studies were positive for ANA, anti-U1-RNP, and anti-SS-A antibodies. Anti-dsDNA, anti-Smith, anti-Scl70, and anti-centromere antibodies were negative. The positive anti-U1-RNP antibodies are the serologic hallmark of MCTD [19]. The clinical phenotype in this case, with swollen digits and esophageal involvement but lacking the classic features of SLE such as malar rash, oral ulcers, or nephritis, was most consistent with a diagnosis of MCTD rather than SLE [19]. The presence of scleroderma features such as swollen hands and gastroesophageal reflux has been shown to be significantly associated with the diagnosis of MCTD in patients with anti-U1-RNP antibodies, whereas the absence of these features suggests an alternative connective tissue disease, particularly SLE [19].

Taken together, the clinical and serologic findings were most consistent with MCTD as the primary etiology of pericardial effusion, though a definitive diagnosis would require longitudinal follow-up and rheumatologic confirmation, while subclinical hypothyroidism and hypoalbuminemia were considered less likely sole explanations given the large volume of the effusion and early tamponade physiology. Nevertheless, a multifactorial contribution from these conditions cannot be entirely excluded.

Several classification criteria sets have been proposed for MCTD, including those by Sharp, Alarcón-Segovia, Kasukawa, and Kahn, with no single universally accepted standard [6]. In a comparative study of 45 patients with anti-U1-RNP antibodies, Alarcón-Segovia's criteria demonstrated the best balance of sensitivity (62.5%) and specificity (86.2%), comparable to Kahn's criteria [20]. The Kasukawa criteria were applied in this case because they incorporate organ-specific features organized by disease category (SLE-like, SSc-like, and PM-like), which best captured this patient's clinical phenotype of pericarditis/serositis and esophageal involvement alongside swollen digits and anti-U1-RNP positivity.

Cardiac involvement is common in MCTD, although often clinically silent. A systematic review of 616 patients with MCTD demonstrated a prevalence of cardiac involvement ranging from 13% to 65%. The most frequent cardiac manifestation was pericarditis, ranging from 30% to 43% in prospective studies [6]. Cardiovascular abnormalities such as pericarditis, mitral valve prolapse, pulmonary hypertension, and coronary artery intimal hyperplasia have been well described [21].

Pericarditis is a recognized feature of MCTD, but it is uncommon as a presenting manifestation of the disease. Pericardial involvement in MCTD is usually preceded by other features of the disease, such as Raynaud's phenomenon, arthritis, or myositis. Large pericardial effusion or cardiac tamponade as the initial presentation of MCTD has been described in only a small number of case reports, including an elderly female with cardiac tamponade as the first manifestation of MCTD [22], a case of pleuritis-pericarditis as the initial presentation [23], and a case of cardiac tamponade in a patient with connective tissue disease [7]. However, the unique contribution of the present case is not just the rarity of pericardial effusion as an initial MCTD manifestation but also the specific diagnostic challenge of separating MCTD from concurrent severe malnutrition and subclinical hypothyroidism. These two competing causes could independently explain the effusion and may have delayed recognition of the underlying autoimmune disease.

The gastrointestinal features in this patient also provide added evidence in support of MCTD. Esophageal involvement is the most frequent gastrointestinal manifestation of MCTD. In a prospective study, heartburn and dysphagia were reported in 48% and 38% of patients, respectively [24]. Esophageal dysfunction in MCTD can manifest as decreased amplitude and coordination of peristaltic waves and reduced lower esophageal sphincter competency, similar to the findings in SSc, but usually less pronounced [25]. This patient was found to have an esophageal stricture on EGD consistent with chronic gastroesophageal reflux. Although esophageal dysmotility is a well-described complication of MCTD, esophageal manometry was not performed in this case, and the attribution of the stricture to MCTD-related dysmotility remains presumptive.

This case warrants discussion of the echocardiographic findings. The large circumferential pericardial effusion with an end-diastolic diameter of 4.87 cm, right atrial systolic collapse, and significant respiratory variation in tricuspid inflow were suggestive of early tamponade physiology. However, the lack of right ventricular diastolic collapse, the most specific echocardiographic finding for cardiac tamponade, and a small, collapsible inferior vena cava suggested that frank tamponade had not yet developed [1]. The hemodynamic effects of pericardial effusion are more related to the speed of fluid accumulation than to the absolute volume, because pericardial compliance can increase with the slow development of effusions [1]. The chronic, insidious nature of the effusion in this patient likely allowed pericardial adaptation such that acute hemodynamic deterioration did not occur despite the large volume of fluid.

Given the large size of the effusion and early signs of tamponade physiology, pericardiocentesis was a reasonable option. The 2025 ACC Expert Consensus Statement advocates for pericardiocentesis in the setting of cardiac tamponade and for diagnostic purposes when there is suspicion of specific etiologies such as bacterial, tuberculous, or malignant pericarditis [1]. In this case, the process was both therapeutic and diagnostic, with evacuation of approximately 1000 mL of clear yellow fluid and analysis, which excluded infectious and malignant etiologies. A limitation of this case is the absence of pericardial fluid chemistry (protein, LDH, glucose) and cell count with differential, which would have aided in further characterizing the effusion as transudative or exudative and narrowing the differential diagnosis.

## Conclusions

In conclusion, this case highlights two specific clinical lessons. First, the noninflammatory phenotype of pericardial disease, characterized by normal CRP and absence of fever, should prompt consideration of autoimmune etiologies, even when nutritional or metabolic causes appear sufficient to explain the effusion. Second, subtle physical examination findings such as swollen digits can serve as critical diagnostic clues that redirect the workup toward an underlying systemic autoimmune disease. While MCTD was considered the most likely primary etiology of the pericardial effusion in this patient, the potential contributions of concurrent malnutrition-related hypoalbuminemia and subclinical hypothyroidism cannot be entirely excluded, and the effusion may have been multifactorial in nature. This case emphasizes the necessity of comprehensive serologic evaluation in patients with idiopathic pericardial effusions and the value of multidisciplinary follow-up to address the multiple organ manifestations of MCTD.
